# A hypoxic signature marks tumors formed by disseminated tumor cells in the BALB-neuT mammary cancer model

**DOI:** 10.18632/oncotarget.8859

**Published:** 2016-04-20

**Authors:** Aichi Msaki, Anna Pastò, Matteo Curtarello, Maddalena Arigoni, Giuseppina Barutello, Raffaele Adolfo Calogero, Marco Macagno, Federica Cavallo, Alberto Amadori, Stefano Indraccolo

**Affiliations:** ^1^ Istituto Oncologico Veneto - IRCCS, Padova, Italy; ^2^ Department of Molecular Biotechnology and Health Sciences, Molecular Biotechnology Center, University of Torino, Torino, Italy; ^3^ Department of Surgery, Oncology and Gastroenterology, University of Padova, Padova, Italy

**Keywords:** breast cancer, Her2/neu, hypoxia, DTC, tumorigenesis

## Abstract

Metastasis is the final stage of cancer progression. Some evidence indicates that tumor cell dissemination occurs early in the natural history of cancer progression. Disseminated tumor cells (DTC) have been described in the bone marrow (BM) of cancer patients as well as in experimental models, where they correlate with later development of metastasis. However, little is known about the tumorigenic features of DTC obtained at different time points along tumor progression. Here, we found that early DTC isolated from BM of 15-17 week-old *Her2/neu* transgenic (BALB-neuT) mice were not tumorigenic in immunodeficient mice. In contrast, DTC-derived tumors were easily detectable when late DTC obtained from 19-22 week-old BALB-neuT mice were injected. Angiogenesis, which contributes to regulate tumor dormancy, appeared dispensable to reactivate late DTC, although it accelerated growth of secondary DTC tumors. Compared with parental mammary tumors, gene expression profiling disclosed a distinctive transcriptional signature of late DTC tumors which was enriched for hypoxia-related transcripts and was maintained in *ex-vivo* cell culture. Altogether, these findings highlight a different tumorigenic potential of early and late DTC in the BALB-neuT model and describe a HIF-1α-related transcriptional signature in DTC tumors, which may render DTC angiogenesis-competent, when placed in a favourable environment.

## INTRODUCTION

Despite advances in early detection of cancer, metastasis still constitutes a major obstacle in treating cancer patients, due to poor understanding of key factors regulating its occurrence [1, 2]. The prevailing view assumes that tumor cells can acquire ability to metastasize in advanced cancer stages [3], but this contrasts with the finding that some early-stage cancer patients already present solitary tumor cells in the bone marrow (BM) [1]. Moreover, the emergence of metastasis years or even decades after apparently successful surgical removal and treatment of primary lesions argues for the reactivation of residual disseminated tumor cells (DTC) [1, 4, 5]. To explain this phenomenon, it has been proposed that after conventional cancer treatments some tumor cells may survive in a state of dormancy lodged in different organs, whereby they are not clinically detected [4]. Thereafter, by as yet unknown mechanisms, these cells switch to a proliferative state and produce a relapse [4, 5]. The identification of factors that regulate tumor dormancy may thus provide new insights for preventing the arising of metastasis.

The presence of DTC in the BM of breast cancer patients has been found to be a strong predictor for local relapse, metastasis and overall survival [6]. A similar correlation has been described even in colo-rectal cancer patients, who rarely develop skeletal metastasis [7]. Further characterization of DTC has revealed that most cells obtained from carcinoma patients are viable and their proliferative capacity is associated with worst prognosis [8]. In contrast, other studies have shown that DTC are heterogeneous, with some displaying fewer chromosomal aberrations compared to the primary tumor counterpart, suggesting that their inability to colonize distant organs is due to their non-fully transformed status [9-11]. Some studies also claimed that most DTC found in the BM exhibit stem cell features [12], while others have proposed that DTC dormancy could result from the balance of surrounding mitogenic and stress signals [13]. Which pre-existing feature or condition endows tumorigenic/metastatic capacity to DTC is still unknown, although it is likely that a combination of both intrinsic (genetic and/or epigenetic) and microenvironmental factors could be at play. Therefore, whilst DTC detection has been accepted as an independent prognostic tool in the clinics, their role in tumor recurrence and metastasis, as well as the underlying mechanisms, is far from clear.

In 2008, Hüsemann *et al.* demonstrated that DTC derived early during tumor progression (early DTC) were tumorigenic [14]. Using the BALB-neuT mouse mammary cancer model, these authors were able to accurately trace the timing of tumor cell dissemination at the earliest signs of atypical ductal hyperplasia in mammary glands of these animals [14]. An important finding of this study was that DTC from the BM of BALB-neuT mice could be reactivated when transplanted into irradiated syngeneic hosts. This suggested that DTC found in the BM have tumorigenic potential and lay dormant till they are released from this state by as yet unknown events. In this scenario, irradiation has been proposed to promote metastatic growth by inducing inflammation and angiogenesis [14-16]. In previous studies, we and others have shown that angiogenesis can lead to escape from tumor dormancy in tumor xenograft models [17, 18]. Given these premises, we sought to further investigate the contribution of angiogenesis in the reactivation of DTC obtained at various stages of tumor progression in the BALB-neuT mouse mammary cancer model and to better characterize DTC-derived tumors.

## RESULTS

### Bone marrow-derived late, but not early, DTC have tumorigenic features

**Figure 1:**
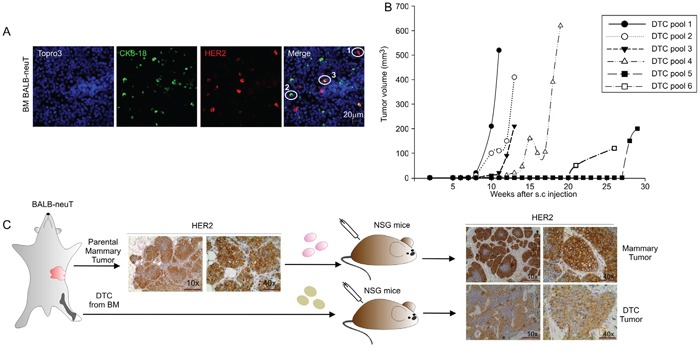
Tumorigenic capacities of BALB-neuT Disseminated Tumor Cells (DTC) **A.** Representative images of BM cells from BALB-neuT mice stained for Her2/*neu* oncogene (HER2) and Cytokeratin 8-18 (CK 8-18). CK8-18 positive cells are shown in green, HER2-positive cells in red and HER2/CK8-18 double-positive cells in yellow; nuclei are stained in blue. In the merge panel: 1, HER2-positive cells; 2, CK8-18 positive cell; 3, HER2/CK8-18 double-positive cell. Scale Bar, 20 μm. **B.** Growth curves of DTC tumors generated from the injection of late DTC from BALB-neuT BM pools. Tumor volumes were plotted as a function of time (weeks) after injection. **C.** Immunohistochemical analysis of HER2 expression in parental mammary tumor and tumors generated from the s.c. injection into NSG mice of BALB-neuT parental mammary tumor cells (mammary tumor, upper panels) and BALB-neuT BM cells (DTC tumor, lower panels). Light microscopy images were taken at 10x magnification (scale bar 200 μm) and 40x (scale bar 50 μm).

In the BALB-neuT transgenic mouse model, mammary cancer growth is driven by the activated rat *Her2/neu* oncogene under the hormone-responsive mouse mammary tumor virus promoter (MMTV) [19]. Identification of DTC in the BM of BALB-neuT mice relies on the detection by immunofluorescence of cells expressing either the epithelial cell marker CK8-18 or the *Her2/neu* oncogene (HER2) or both markers [14] (Figure 1A). To test the tumorigenicity of DTC derived from the BM of BALB-neuT mice, 5 × 10^6^ BM-derived cells were injected sub-cutaneously (s.c.) into immunodeficient female NOD-SCID-γ^−/ −^ (NSG) mice. Eleven-to-seventeen week-old BALB-neuT mice bearing pre-invasive lesions (including atypical hyperplasia and *in situ* carcinoma), were arbitrarily categorized as ‘early DTC donors’ (young BALB-neuT), whereas 19-to-22 week-old BALB-neuT mice bearing invasive carcinomas were classified as ‘late DTC donors’ (old BALB-neuT). As summarized in Table 1, we did not observe any tumor in mice inoculated with early DTC (0/38 injection sites) over an eight-month observation period. On the contrary, injection of 16 out of 36 (44%) late DTC-containing BM cells gave rise to tumors (DTC tumors) (Table 1). No obvious correlation was seen between the estimated number of DTC present in the *inocula* and tumor generation (Table 1). Moreover we did not find statistically significant differences between late and early DTC in the expression of the CK8-18 and/or HER2 markers (Table 1). In these experiments, we initially inoculated pools of BALB-neuT BM cells. Indeed, we observed a great variability in the timing of tumor development (Figure 1B), but donor variability in late DTC content did not apparently affect DTC tumorigenicity (Table 1). We therefore decided to use single DTC donor mice, bearing different tumor burden, and sought a correlation between tumor outgrowth, DTC contents, and time for tumor development. As shown in Supplementary Table S1, when BM cells from individual mice were used, we did not find any correlation between tumor burden (defined by total tumor volume and number of tumors) and DTC content, DTC phenotype or the tumorigenic behaviour of DTC in recipient mice.

**Table 1:** Tumorigenic capability of early and late Disseminated Tumor Cells (DTC) in NSG mice^a^

	Donor Age	DTC injected HER2+	DTC injected CK8-18+	DTC injected HER2/CK8-18+	Total no. of DTC injected^b^	Tumors/injected sites	Tumor take (%)
**Early DTC donor**	15 weeks	10	0	5	15	0/4	
	15 weeks	17	3	0	20	0/4	
	15 weeks	25	5	0	30	0/4	
	15 weeks	20	0	20	40	0/2	
	15 weeks	15	15	25	55	0/2	
	15 weeks	35	15	20	70	0/2	
	15 weeks	45	20	30	95	0/2	
	17.5 weeks	n.d	n.d	n.d	n.d	0/9	
	17.5 weeks	n.d	n.d	n.d	n.d	0/9	
					**Total**	**0/38**	**0**
**Late DTC donor**	20 weeks	3	6	3	12-15	4/12	
	20 weeks	5	14	2	21-22	1/8	
	20 weeks	20	0	5	20-30	3/6	
	20 weeks	30	0	6	30-40	6/8	
	19 weeks	15	35	0	50	2/2	
					**Total**	**16/36**	**44**

a) BM pools from BALB-neuT mice of various ages were harvested, and inoculated into the s.c. pocket of NSG mice (5 × 10^6^ BM-derived cells/site). Mice were inspected weekly and tumors scored when volume exceeded 10 mm^3^.

b) DTC number was estimated by staining for CK8-18 and HER2 antigens.

### Late DTC tumors recapitulate parental BALB/neuT mammary tumors

DTC tumors expressed HER2 similarly to parental BALB-neuT mammary tumors (parental mammary tumor) and to BALB-neuT mammary tumor cells grown s.c. in NSG mice (mammary tumor; Figure 1C). Flow cytometry analysis confirmed these data and showed that HER2 expression in cells recovered from the DTC tumor was of similar intensity to the parental mammary tumor cells and TUBO cells, a cell line derived from a BALB-neuT tumor (Supplementary Figure S1A). Moreover, cells recovered from DTC tumors could re-generate new tumors in immunodeficient as well as immunocompetent syngeneic mice (Table 2). To test whether angiogenic factors could positively affect DTC outgrowth, we initially tested the ability of two major angiogenic factors, bFGF and VEGF (vascular endothelial growth factor), in promoting blood vessel formation in our setting. BM-derived cells were s.c. injected in Matrigel sponges with or without the angiogenic factor bFGF, VEGF alone or the combination of the two. As shown in Supplementary Figure S2A, only in the case of BM-derived cells injected with the bFGF or a combination of bFGF/VEGF we could clearly observe blood vessel formation. Moreover, these angiogenic factors, alone or in combination, did not change the frequency of tumor development following injection of DTC-containing BM samples, as shown in Supplementary Figure S2B. We therefore set out a new experiment to determine whether bFGF could accelerate the outgrowth of late DTC. Thus BM-derived cells were injected s.c. into Matrigel supplemented with bFGF. After a 30-week observation period, comparable frequencies of tumors were observed in BM-derived cells containing late DTC injected with or without bFGF (Supplementary Table S2), suggesting that exogenous angiogenic factors are not needed to reactivate late DTC. In any case, bFGF was able to accelerate the growth of secondary DTC tumors (Supplementary Figure S1B). The same experiment was also performed by injecting early DTC in the presence or absence of bFGF; however, the presence of this angiogenic factor did not modify the tumorigenicity of early DTC and no tumor outgrowth was observed (data not shown).

**Table 2:** DTC tumors can be serially transplanted into both immunodeficient and immunocompetent syngeneic mice^a^

	NSG mice		BALB/c mice	
	Tumors/site inoculated	Tumor take (%)	Tumors/site inoculated	Tumor take (%)
**DTC Tumor cell line 1**	12/12	100%	7/8	96.8%
**DTC Tumor cell line 2**	4/4	100%	8/8	100%
**DTC Tumor cell line 3**	4/4	100%	8/8	100%
**Mammary Tumor cell line**	4/4	100%	8/8	100%

a) Cells derived from DTC (DTC tumor cell line) or mammary tumors (mammary tumor cell line) were s.c. inoculated into immunodeficient NSG or immunocompetent syngeneic BALB/c mice. Mice were inspected weekly and tumors scored when volume exceeded 10 mm^3^.

### Gene expression profiling identifies HIF-1α pathway deregulation in DTC tumors

**Figure 2:**
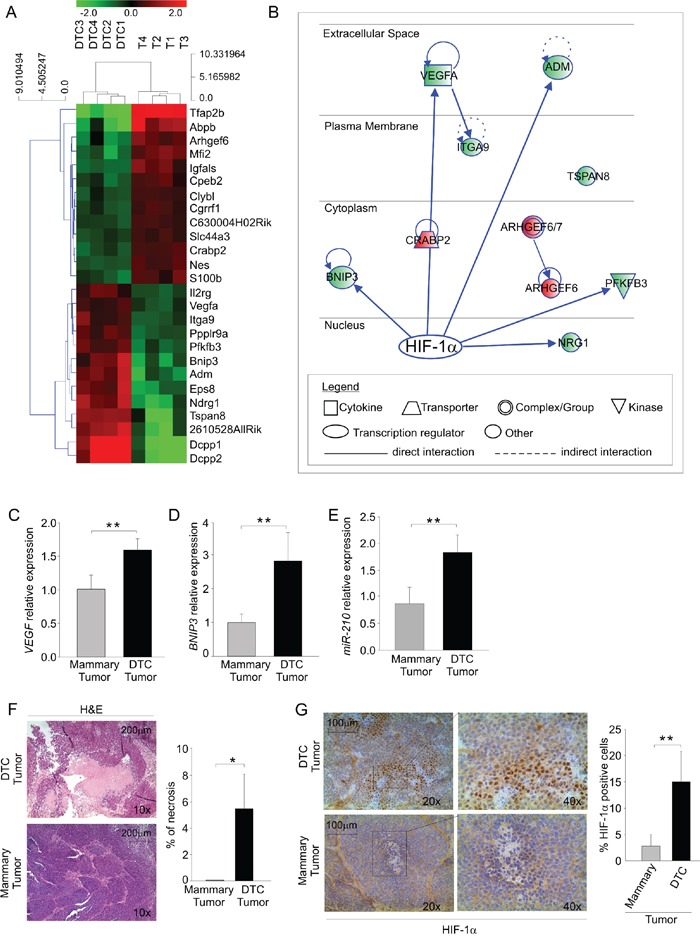
DTC tumors display enhanced expression of hypoxia-regulated genes **A.** Heat map of differentially regulated genes; the green color depicts genes that are downregulated and the red denotes genes that are up-regulated in DTC tumors (DTC 1-4) and mammary tumors (T 1-4). **B.** Ingenuity pathway analysis (IPA) illustrating HIF-1α as a possible master regulator of differentially regulated genes in DTC tumors compared to mammary tumors. Up-regulated and down-regulated genes are shown in red and in green, respectively. **C-D-E.** *VEGF* C. and *BNIP3* D. mRNA expression and *miR-210* quantification E. in mammary and DTC tumors. The histograms depict relative expression compared to mammary tumor expressed as fold change. Data are expressed as mean values (± SD) of 4 different samples/group. **F.** Histopathologic evaluation of necrotic areas in mammary and DTC tumors. Representative haematoxylin and eosin-stained sections (H&E) of each tumor type were photographed on the left at 10x (scale bar 200 μm). The right histogram illustrates the quantification of necrotic areas over total tumor area. Data are expressed as mean values (± SD) of 6 different samples/group. **G.** Immunohistochemical analysis of HIF-1α expression in frozen sections of mammary and DTC tumors. A representative image of each tumor stained for HIF-1α is shown on the left at 20x; the quadrant indicates the area photographed at 40x (scale bar 100 μm). The right histogram shows the quantification of HIF-1α positive nuclei; data are expressed as mean values (± SD) of 5 different samples/group.*P < 0.05, **P < 0.01.

To further characterize DTC tumorigenic behavior, we next compared gene expression profiles in DTC tumors and mammary tumors grown in the s.c. tissue of NSG mice (see Figure 1C). We analyzed genes differentially regulated in four DTC tumors and four mammary tumors. As shown in Figure 2A, bioinformatics and microarray analysis of mRNA expression profile revealed 26 top genes differentially regulated between DTC and mammary tumors. These include several relevant genes involved in the growth, proliferation and survival of cancer cells (Table 3). Furthermore, Ingenuity pathway analysis (IPA) identified relevant signalling networks activated and up-regulated in DTC tumors compared to mammary tumors (Figure 2B). Despite comparable tumor volume size (data not shown), DTC tumors showed increased levels of hypoxia-regulated genes such as the cytokine *VEGF* [20], *Adm* (adrenomedullin) [21], *Bnip3* (BCL2/Adenovirus E1B 19kD-Interacting Protein 3) [22], *Pfkfb3* (6-phosphofructo-2-kinase/fructose-2,6-biphosphatase 3) [23], *Itga9* (Integrin alpha-9) [24], and *NRG1* (neuregulin 1) [25] (Figure 2B). The mRNA expression of *VEGF* and *BNIP3* was also validated by qPCR. As shown in Figure 2C, DTC tumors expressed significantly more *VEGF*, compared to mammary tumors; however, this finding did not translate into differences in microvessel density (MVD) between the two types of tumors (data not shown). *BNIP3* expression also was up-regulated in DTC tumors compared to mammary tumors (Figure 2D) as well as the hypoxia-regulated miRNA, *miR-210* [26] (Figure 2E). Since *VEGF* and *BNIP3* contain HIF-1α responsive elements (HRE), we inferred that DTC tumors could be more hypoxic. In fact, DTC tumors had larger necrotic areas compared to mammary tumors (Figure 2F). Furthermore, a significantly higher HIF-1α nuclear accumulation was seen in DTC tumors (Figure 2G). Altogether, these observations suggest that DTC tumors exhibit a relatively higher hypoxic phenotype than mammary tumors.

**Table 3:** Top differentially regulated genes between mammary tumors and DTC tumors^a^

Gene Symbol	log_2_ Fold Change mammary vs. DTC tumor	*P* Value
Tfap2b	4.00	0.00004
Abpb	2.66	0.02277
Mfi2	1.72	0.01500
Arhgef6	1.62	0.03335
Igfals	1.54	0.01500
S100b	1.27	0.03968
Crabp2	1.27	0.02177
Nes	1.26	0.01500
Cpeb2	1.18	0.04957
C630004H02Rik	1.07	0.02490
Cgrrf1	1.06	0.03335
Clybl	1.04	0.04957
Slc44a3	1.02	0.03936
Vegfa	−1.03	0.04872
Itga9	−1.21	0.03968
Il2rg	−1.24	0.02322
Ppp1r9a	−1.36	0.01500
Pfkfb3	−1.42	0.01808
Bnip3	−1.87	0.03936
Eps8	−1.95	0.01808
Adm	−2.00	0.01808
Ndrg1	−2.28	0.01500
Tspan8	−2.30	0.04836
2610528A11Rik	−2.51	0.04746
Dcpp1	−3.93	0.01500
Dcpp2	−4.59	0.02490

a) RNA from DTC tumors and mammary tumors where used to hybridize Affymetrix chips. Top differentially expressed genes were calculated using cut-off of 0.05 *P* value with the Bonferroni correction method. The table lists differentially expressed genes (gene symbols), their magnitude expressed as fold change of mammary vs. DTC tumor expression level (Log_2_ Fold Change) and the False Discovery Rate (FDR) *P*-value score.

### Cells derived from DTC tumors maintain their hypoxic phenotype upon *in vitro* passage

**Figure 3:**
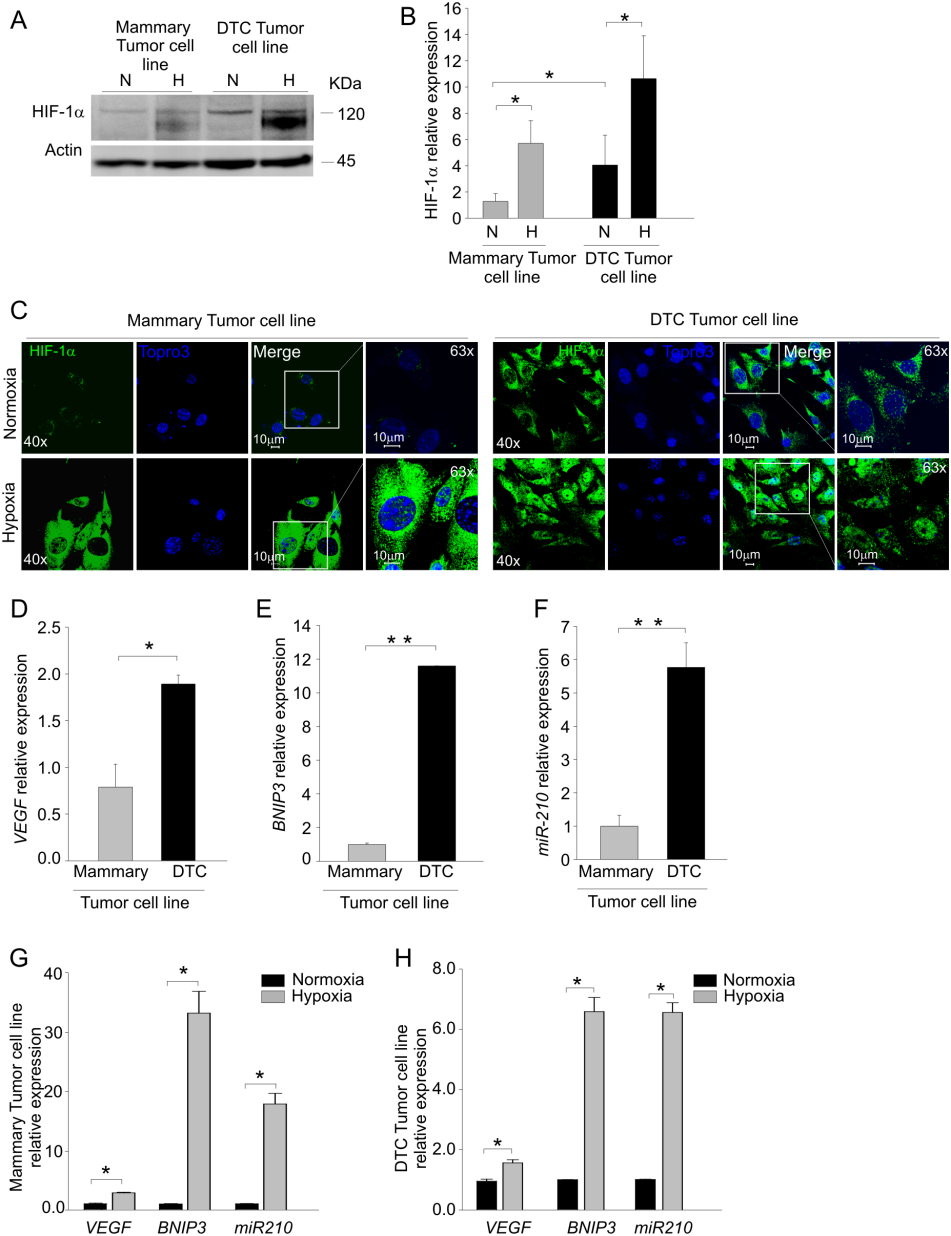
DTC tumor cells maintain *in vitro* up-regulation of HIF-1α expression **A.** Western blot analysis of HIF-1α protein levels in mammary and DTC tumor cell lines cultured under normoxic (N) or hypoxic (H) conditions. The molecular weight of HIF-1α and Actin, used as a loading control is indicated on the right. **B.** Quantification of HIF-1α protein expression by densitometry. The histograms represent quantification of HIF-1α normalized to individual actin levels, and relative to HIF-1α protein levels in normoxia of mammary tumor cell line. **C.** Immunofluorescence analysis of HIF-1α localization in mammary and DTC tumor cell lines in normoxic (top row) and hypoxic conditions (bottom row). In green HIF-1α staining, in blue nuclei. Images acquired at 40x and 63x magnification, scale bar 10 μm. **D-E-F.** qPCR analysis of *VEGF* D. and *BNIP3* E. mRNA levels and *miR-210* expression F. in cells derived from mammary and DTC tumor cell lines. **G-H.** qPCR analysis of *VEGF*, *BNIP3* and *miR-210* levels following incubation under hypoxia in mammary G. and DTC tumor cell lines H. Data are presented as relative to level under normoxic conditions. All data represent mean values ± SD. *P < 0.05, **P < 0.01.

To discern whether this hypoxic signature was imposed by the microenvironment or it was dependent on intrinsic features of tumor cells, we next investigated HIF-1α levels in the cells isolated from DTC tumors. After short *in vitro* expansion (2 to 4 passages) of the cells derived from a DTC tumor (DTC tumor cell line) and mammary tumor (Mammary tumor cell line) we evaluated protein levels of HIF-1α by Western blotting. Under normoxic culture conditions, we found almost four times more HIF-1α protein levels in the DTC-tumor cell line compared to the mammary tumor cell line, in which HIF-1α was barely detectable (Figure 3A-3B). As expected, HIF-1α accumulated in response to hypoxia in both cell types (Figure 3A-3B). The cells maintained this phenotype for several *in vitro* passages (data not shown). We also evaluated the intra-cellular distribution of HIF-1α by immunofluorescence in normoxia and hypoxia (Figure 3C). Whilst both cell lines in response to hypoxia showed nuclear localization of HIF-1α, we found also an intense cytoplasmic HIF-1α signal in DTC tumor cell line in normoxia (Figure 3C). In contrast, HIF-1α was barely detectable in the cultures from the mammary tumor cell line (Figure 3C). We next examined whether gene expression levels of *VEGF* and *BNIP3* transcripts were affected. As shown in Figure 3D, *VEGF* mRNA levels of the DTC tumor cells almost doubled compared to mammary tumor cells. The mRNA levels of *BNIP3* also were markedly up-regulated in DTC compared to mammary tumor cell line (Figure 3E). In support to these results, also *miR-210* was considerably up-regulated in the DTC tumor cell line (Figure 3F). As expected, these transcripts were strongly upregulated in each cell line following exposure to hypoxia (Figure 3G and 3H). Altogether, these findings indicated that cells derived from DTC tumors stably maintain *in vitro* a hypoxic phenotype.

### HIF-1α is required for DTC tumorigenic potential

**Figure 4:**
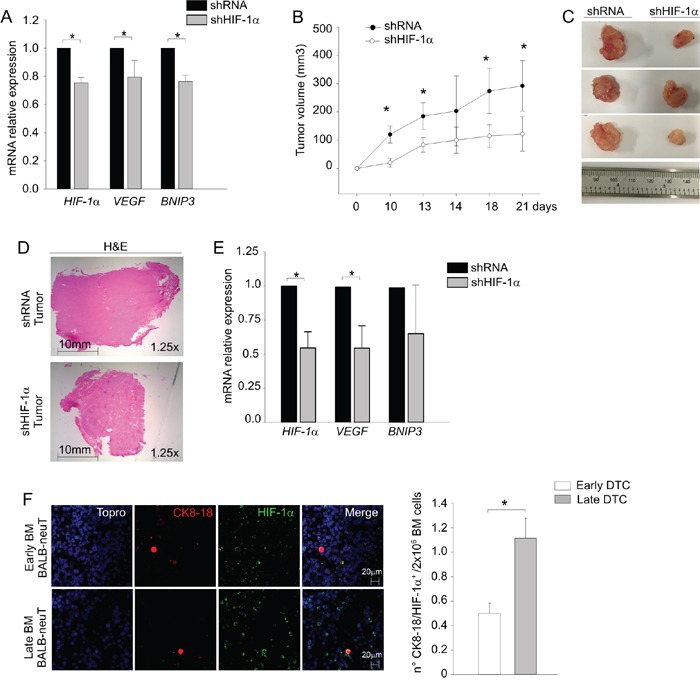
HIF-1α is required for DTC tumorigenicity **A.** *HIF-1α*, *VEGF* and *BNIP3* mRNA levels were measured by qPCR in DTC transduced with a lentiviral vector silencing HIF-1α (shHIF-1α) compared to control (shRNA). **B.** Tumor growth curves of control (shRNA, black dots) and HIF-1α (shHIF-1α, white dots) transduced DTC cells. Mean tumor volume (n=6 samples/group) ± SD was plotted as a function of time (days). **C.** Representative pictures of tumors at sacrifice of the mice (day 21). **D.** Representative haematoxylin and eosin-stained sections (H&E) of tumors. Pictures were acquired at 1.25x magnification, scale bar 10 mm. **E.** Analysis of *HIF-1α*, *VEGF* and *BNIP3* mRNA levels in shRNA and shHIF-1α DTC tumors by qPCR. **F.** HIF-1α expression in early and late DTC by immunofluorescence analysis. On the left, representative confocal images of early (upper panels) and late (lower panels) DTC taken at 63x magnification, scale bar 20 μm. Nuclei were stained in blue, CK8-18 in red and HIF-1α in green. On the right, histogram reporting mean numbers of CK8-18^+^/HIF-1α^+^ DTC per 2 × 10^6^ BM cells. *P < 0.05.

To determine whether this hypoxic signature was necessary for DTC tumor growth, we silenced HIF-1α in DTC by lentiviral vectors expressing HIF-1α-specific short hairpin (sh) RNA and monitored their growth in the s.c. pocket of NSG mice (Figure 4A-4E). Notwithstanding a modest (20-30%) reduction in the mRNA levels of HIF-1α and its downstream target genes *VEGF* and *BNIP3* (Figure 4A), we observed significant differences in tumor growth between silenced DTC and control cells (Figure 4B). As shown in Figure 4C, HIF-1α-silenced DTC cells (shHIF-1α) formed smaller tumors compared to control (shRNA) cells. The recovered tumors were morphologically similar to controls, as shown by H&E staining (Figure 4D) and maintained downregulated expression of HIF-1α and its two target genes *VEGF* and *BNIP3* (Figure 4E).

Since we previously observed differences in early and late DTC tumorigenicity we evaluated HIF-1α expression in BM samples containing early or late DTC. As shown in Figure 4F, the BM of late DTC donors contained a significantly higher number of CK8-18^+^/HIF-1α^+^ DTC. Altogether, these results suggest that HIF-1α plays a role in the outgrowth of DTC tumors.

### DTC tumors and metastasis tumors partly share the hypoxic phenotype

**Figure 5:**
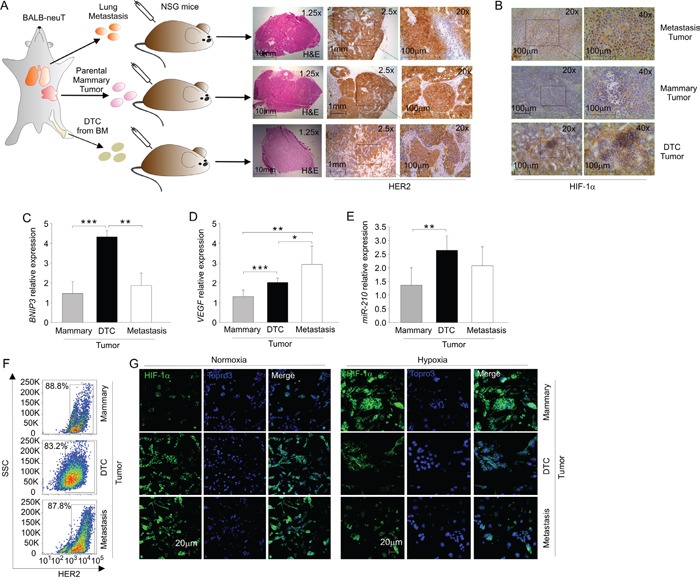
DTC-tumor hypoxic profile is partly shared with BALB-neuT tumor metastasis **A.** Schematic representation of mammary, DTC and metastasis tumor generation; the right panel shows the histopathological analysis of mammary, DTC or metastasis tumors. A representative of each tumor frozen section was stained with H&E and HER2. Light microscopy images were taken at a magnification 1.25x (scale bar 10 mm), 2.5x (scale bar 1 mm) and 20x (scale bar 100 μm). **B.** Immunohistochemical analysis of HIF-1α expression in metastasis, mammary and DTC tumors. A representative tumor frozen section is shown, photographed at 20x and at 40x magnification. **C-D.** qPCR analysis of mRNA levels of *BNIP3* **C.** and *VEGF* **D.** in mammary, DTC and metastasis tumors. Data represent mean relative expression of mRNA compared to mammary tumor (± SD), expressed as fold change. **E.** qPCR analysis of *miR-210* expression in mammary, DTC and metastasis tumors. Data are expressed as mean values (± SD), relative to the level of *miR-210* in mammary tumors. **F.** Flow cytometric analysis of HER2 expression in mammary, DTC and metastasis tumor cell lines. A representative plot of the percentages of HER2^+^ cells in the population of mammary, DTC and metastasis tumor cell lines is shown. **G.** Immunofluorescence analysis of HIF-1α localization in mammary, DTC and metastasis tumor cell lines in normoxic and hypoxic conditions. In green HIF-1α staining, in blue nuclei; scale bar 20 μm. *P < 0.05, **P < 0.01, ***P < 0.001.

To compare the phenotype of DTC tumors and BALB-neuT lung metastasis, we generated s.c. tumors by injecting NSG mice with metastatic cells dissociated from the lungs of 28-week-old BALB-neuT mice, according to the experimental layout shown in Figure 5A. As shown in Figure 5A (top row), similarly to mammary tumors (middle row) and DTC tumors (bottom row), metastasis tumors almost exclusively contained HER2^+^ cells. However, tumors formed by metastasis-derived cells showed a pattern of HIF-1α expression more similar to DTC-derived tumor than mammary, as shown in Figure 5B. We next analysed by qPCR the levels of HIF-1α target genes whose expression was relatively high in DTC tumors, including *VEGF* and *BNIP3*, as well as *miR-210*. We found that DTC tumors expressed significantly higher *BNIP3* levels compared to both mammary and metastasis tumors (Figure 5C). However, metastasis tumors expressed higher mRNA level of *VEGF* compared to mammary and DTC tumors (Figure 5D). On the other hand, metastasis tumors did not significantly differ in *miR-210* levels compared to DTC tumors (Figure 5E). We also evaluated stabilization of HIF-1α in the cells recovered from metastasis tumors (Figure 5F-5G). We found that although metastasis tumor cell lines equally expressed HER2 compared to the mammary and DTC tumor cell lines (Figure 5F), they showed higher HIF-1α levels compared to DTC tumor cell line (Figure 5G, left panel). These findings suggest that metastasis tumors might have an exacerbated, hypoxic phenotype compared to DTC tumors. Furthermore, HIF-1α in these cells relocated, as expected, in the nuclei when placed under hypoxic conditions (Figure 5F, right panel). In conclusion, the HIF-1α signature detected in DTC tumors is substantially shared by tumors formed by metastatic cells found in the lung of BALB-neuT mice.

## DISCUSSION

Many studies characterized DTC by analyzing lesions derived from the injection of established cell lines or primary tumor cells as a source of DTC [27, 28]. In addition, most clinical studies reported a strong correlation between DTC presence and development of future metastases, even in patients with early stage tumors. However, only a limited number of studies directly addressed the tumorigenic capability of DTC or circulating tumor cells (CTC), and in all cases they utilized samples from patients with advanced cancers [29-31].

We were intrigued by the finding that early dormant DTC derived from the BALB-neuT transgenic mice could be reactivated to invade the BM by events following host irradiation [14]. This suggested that early DTC could be dependent on microenvironment cues to acquire tumorigenic properties, such as angiogenesis or inflammation. Therefore, the initial purpose of this study was to better characterize the tumorigenic behavior of early DTC in the context of pro-angiogenic microenvironment.

Whilst this study did not find early DTC to be tumorigenic, we found that about 44% of BM samples containing late DTC were tumorigenic (Table 1). We found DTC tumorigenicity to be independent from the number of injected DTC (Table 1) and on the tumor burden of the donor (Supplementary Table S1), whereas it correlated with the age of the DTC donor mice. Hypothetically, subtle genetic aberrations or epigenetic alterations might underlie the different tumorigenic capability, as gross chromosomal differences between DTC from different stages of tumor in the BALB-neuT mouse model were not detected in previous studies [14]. It is still unclear whether early DTC could evolve into late DTC or if late DTC originate from the dissemination of cells from later stages of primary tumors. In support of the latter idea, DTC obtained from patients with clinically detectable tumors were found to be highly malignant [32]. Moreover, so far only CTC derived from patients with advanced stage cancer have shown tumorigenic potential in immunodeficient mice [29-31].

With regard to the finding that only 44% of BM samples from late DTC donors were tumorigenic, it should be stressed that DTC, like primary tumor cells, have been described to be heterogeneous [9] and therefore it can be presumed that DTC do not have equivalent tumorigenic capabilities. Speculatively, it could well be that not all DTC in this mouse model express the activated *Her2/neu* oncogene at all times, as DTC detection relies on the enumeration of CK8-18 positive cells [14]. Podsypanina *et al*. demonstrated that even untransformed epithelial cells can circulate and settle in a variety of organs for a long time, but only upon expression of the driver oncogene they begin to expand and establish metastatic growth [11]. Activation of oncogenes, such as *Her2/neu*, may be crucial to tip the balance between active growth and dormancy. Indeed, *Her2/neu* activation leads to AKT and ERK phosphorylation [13, 33, 34].

With regard to angiogenesis, our results indicate that the tumorigenic behavior of late DTC is substantially independent from exogenous angiogenic factors, such as bFGF. On the other hand, in line with the established role of angiogenesis as tumor promoter, exogenous bFGF accelerated the growth of secondary DTC tumors. Thus, late DTC appear to be fully equipped with the capability of inducing the angiogenic switch and results of transcriptome analysis lend support to this hypothesis. In fact, microarray data disclosed that DTC tumors have a deregulated hypoxia pathway and that this profile is maintained upon *in vitro* culture of these cells (Figure 2 and 3). We found that DTC tumors and their *ex vivo* culture have elevated mRNA levels of *BNIP3*, a pro-survival gene involved in hypoxia-driven autophagy [22], and *VEGF*, a potent angiogenic factor [20] (Figure 2C-2D and Figure 3D-3E), as well as *miR-210*, a key microRNA that regulates angiogenesis and survival in response to hypoxia [26]. These findings might likely explain why late DTC outgrowth was not affected by the angiogenic factor bFGF (Supplementary Table S2) and also underscore reduced dependence of late DTC from the microenvironment, compared with early DTC. It could thus be that the lack of tumor formation by early DTC is due to an inadequate microenvironment in our experimental setting.

Another interesting observation of our study is that cells derived from DTC tumors maintain high basal levels of HIF-1α protein compared to the mammary tumor cell line (Figure 3A-3C). Some authors found that HIF-1α stabilization is caused by HER2 overexpression [34]. However, we did not find increased HER2 expression in DTC tumors, compared with mammary tumors (Figure 1A-1B), nor did we find significant differences in HIF-1α mRNA levels, implying post-transcriptional stabilization of HIF-1α by as yet unknown mechanisms.

The precise role of HIF-1α in DTC is unknown. Intriguingly, DTC have been described to have predilection for the hematopoietic stem cell niches, a predominantly hypoxic environment [35, 36]. Speculatively, activation of HIF-1α could be critical for DTC to survive in these bone marrow niches or, alternatively, oxygen-independent upregulation of HIF-1α could arise from upregulation of upstream signaling pathways involved in tumor progression. In any case, attenuation of HIF-1α expression was able to reduce tumorigenic capabilities of DTC cells (Figure 4), thus underscoring its contribution to tumor formation. In line with our findings, in the MMTV-PyMT murine model, HIF-1α was shown to be necessary for acceleration of tumor onset and progression [37-40].

Along this line, we also report that metastasis tumors partly shared the hypoxic profile of DTC tumors (Figure 5B-5G). Metastasis tumors did not significantly differ in *miR-210* expression compared to DTC tumors (Figure 5E), but had more pronounced *VEGF* expression (Figure 5D). On the other hand, DTC tumors appeared to have elevated *BNIP3*, compared to the metastasis tumors (Figure 5C). Hypoxia not only promotes cancer dissemination and stemness, but it has also been described to promote metastasis from bone to lung [37, 41, 42], raising the hypothesis that lung metastasis could stem from BM-derived DTC.

Besides a hypoxic profile, DTC tumors also have marked expression of cancer relevant genes (Table 3) such as the metastasis-promoting tetraspanin 8 (*TSPAN8*) [43], proliferation and migration promoting epidermal growth factor substrate 8 (*EPS8*) [44, 45]. These results suggest that the DTC tumor phenotype could endow them with enhanced aggressiveness; indeed, it would be interesting to examine whether this phenotype might constitute a prerequisite for metastasis formation in this mouse model. Thus, our findings also suggest the alluring possibility that late DTC could be enriched in metastasis precursors/metastasis-initiating cells. In future studies, it will be important to refine methods of isolation and characterization of late DTC from the BM, whose number is extremely low, to validate the signature obtained from DTC-derived tumors.

In conclusion, our observations connect tumorigenic features of DTC with a hypoxic signature and prompt further investigation in DTC or CTC from patients.

## MATERIALS AND METHODS

### Transgenic mice

Founder BALB-neuT male mice were kindly provided by Dr. Guido Forni (Torino, Italy) [55]. BALB-neuT mice were bred with in house BALB/c female mice. Female offspring were then screened for the presence of *Her2/neu* oncogene as previously described [55]. NOD/SCID-γ^−/ −^ (NSG) mice were purchased from Charles River and bred in house. Procedures involving animals and their care were performed according to institutional guidelines that comply with national and international laws and policies (EEC Council Directive 86/609, OJ L358, 12 December 1987), and this study was approved by the Institutional Ethics Committee for Animal Studies.

### Cell culture conditions

TUBO cells derived from BALB-neuT mammary cancer cells [46] were maintained in DMEM with 1% Penicillin-Streptomycin (Lonza, Basel, Switzerland), 1% L-Glutamine (Invitrogen, Monza, Italy) and 20% Fetal Calf Serum (FCS, Gibco, Invitrogen), unless otherwise stated. BM cells were harvested by flushing the femurs and tibiae with cold PBS and filtering through 100 μm cell strainers. Red blood cells were lysed by NH_4_Cl/KHCO_3_/EDTA buffer; cells were then re-suspended in PBS and viability assessed by trypan blue dye (Invitrogen) exclusion. For hypoxia experiments, cells were seeded at 5 × 10^5^ cell/well and maintained in either normoxia or hypoxia (0.5 % O_2_) for 24 hours in an *InVivo2 300* hypoxic chamber (Ruskinn Technology, Mid Glamorgan, U.K.). In order to attenuate HIF-1α expression, 3×10^5^ DTC cells were plated and infected overnight with a pLKO1-puro lentivirus encoding an HIF-1α-specific shRNA. Control cells were infected with a vector encoding a shRNA of irrelevant sequence. The following shRNA sequence was used to target HIF-1α:

CCGGTGCTCTTTGTGGTTGGATCTACTCGAGTAGATCCAACCACAAAGAGCATTTTT

Lentiviral plasmids were purchased from Sigma-Aldrich (St. Louis, MO, USA) and the vector was produced as previously described [47].

### Immunofluorescence analysis

To evaluate DTC numbers, BM cells were resuspended in PBS and placed on positively charged slides to air dry. Dried spots of cells were fixed and permeabilized for 15 min with ice-cold methanol at -20°C and then processed for staining. Anti-HER2/ErbB2 (29D8) rabbit monoclonal antibody (Cell Signaling, Boston, MD, USA) was used at 1:400 dilution overnight at 4°C. Guinea-pig anti-Cytokeratin (CK) 8-18 (Progen, Heidelberg, Germany) was used at 1:100 for one hour at room temperature. Anti-HIF-1α rabbit antibody (NB100-479) was purchased from Novus Biologicals (Littleton, CO, USA) and used at 1:100 dilution overnight at 4°C. Alexa Fluorescent secondary antibodies were purchased from Molecular Probes (Invitrogen). Cell nuclei were visualized with TO-PRO-3 (Invitrogen). All images were taken with Zeiss LSM510 (Oberkochen, Germany) and Leica LM5 (Wetzlar, Germany) confocal microscope at the specified magnification. TUBO cells cultured under normoxic or hypoxic conditions (0.5% pO_2_ for 24 h) were used as positive control for HER2 and HIF-1α staining, respectively.

### Immunohistochemical analysis

For immunohistochemical analysis of HER2 expression, tumors were fixed in formalin and included in paraffin. Tumor sections were then de-paraffinized and rehydrated before antigen retrieval in EDTA buffer (pH 8.0). For analysis of HER2 expression on frozen tumor sections, these were fixed in 4% formaldehyde for 10 min. To analyze the expression of HIF-1α, frozen sections were obtained and fixed in ice-cold methanol for 15 min. Endogenous peroxidases were blocked with 3% hydrogen peroxidase treatment for 30 min. To prevent non-specific antigen binding, sections were incubated with 5% normal goat serum 60 min prior to staining with HER2/ErbB2 antibody (1:400; Cell Signaling) or with anti-HIF-1α (Novus Biologicals) 1:200 overnight. The slides were then incubated with the biotinylated secondary antibody, followed by incubation in an avidin-biotinylated peroxidase complex reagent (Vectastain Rabbit ABC Elite kit; Vector Laboratories, Burlingame, CA, USA), and visualized with diaminobenzidine tetra-hydrochloride (DAB; Sigma-Aldrich) treatment. The slides were counterstained with Mayer's haematoxylin, dehydrated, and finally mounted with Entellan (BDH Ltd, Poole, Canada). For HER2 staining, tumor sections from BALB-neuT tumors served as positive control. For HIF-1α staining, sections of hypoxic tumor xenografts analysed in previous studies [48] were used as positive control. Quantification of the percentage of HIF-1α positive nuclei was performed on regions of interest containing approximately 20,000 nuclei, and analysed with Visiopharm software (Horsholm, Denmark).

### Flow cytometry (FACS) analysis

For HER2 staining, cells were fixed with 4% paraformaldehyde and incubated overnight in methanol. The cells were thereafter incubated with 1% BSA to prevent non-specific binding and with anti-HER2/Erb2 (1:400) antibody followed by the appropriate secondary antibody (Alexa 1:500; Invitrogen) [49]. The samples were analysed with FACS LSRII (BD Bioscience, Franklin Lakes, NJ, USA); data were collected from at least 1 × 10^5^ cells/sample and elaborated with FlowJo software (TreeStar, Ashland, OR, USA).

### *In vivo* studies

For subcutaneous (s.c.) injection, 10 × 10^6^ BM cells or 1 × 10^5^ tumor cells were re-suspended in a total of 200 μl PBS with 1 μg murine bFGF (Peprotech, Rocky Hill, NJ, USA) mixed with 400 μl of Matrigel (Becton-Dickinson), according to a previously published protocol [18]. We injected a total of 300 μl (cells plus Matrigel mix) per dorsolateral flank. Tumors were inspected weekly and measured with caliper. Tumor volumes were calculated according to the following formula: Tumor volume (mm^3^) = L (longest diameter) × l (shortest diameter)^2^ × 0.5 [50]. Tumor growth was scored when tumor volumes exceeded 10 mm^3^.

For the Matrigel assay, a total of 5 × 10^6^ of BM cells in 400 μl of Matrigel supplemented with bFGF and/or VEGF were s.c. injected as previously described [18]. After one week Matrigel plugs were recovered, snap frozen in liquid nitrogen and stored at −80°C.

For *in vivo* silencing experiments, DTC cells transduced with lentiviral vectors where s.c injected (2.5×10^5^/flank) in 200 μl of Matrigel in NSG mice.

### Microarray

MoGene 2.0 ST array (Affymetrics, Santa Clara, CA, USA) was used according to manufacturer's instruction using the recommended amount of RNA (100 ηg). Briefly, tumor masses from primary BALB-neuT mammary tumors and DTC tumors grown in NSG mice were snap-frozen at harvest. Cryostat shavings were used to extract RNA and a representative section was kept for Haematoxylin and Eosin (H&E) staining in order to evaluate tumor tissue anatomy and to quantify necrotic areas. The quality and quantity of total RNA from samples was determined using Agilent 2100 Bioanalyzer and Nanodrop spectrophotometer. Hybridization, washing, staining and scanner procedures were done using a Genechip Affymetrix station (Fluidics station 450, GeneChip Scanner 3000) as recommended by manufacturer. Laser scanning generated digitalized image data and CEL files that were used for the subsequent statistical analysis. The data were loaded in oneChannelGUI [51], normalized using sketch-quantile normalization and filtered by IQR > 0.25 to remove unchanged transcripts. To assess differential expression, we used an empirical Bayesian method [52] together with a false discovery rate (FDR) correction of the P-value. Genes were selected as differentially expressed if characterized by a FDR ≤ 0.05 and an absolute log_2_ fold change ≥ 1. The microarray data have been deposited in the GEO database under accession number GSE71251.

### Micro RNA assay

A total of 10 ng of RNA was retro-transcribed with Custom TaqMan Small RNA Assay, using TaqMan MicroRNA Assay primers for miR-210 and snoRNA-202 (all from Applied Biosystems, Foster City, CA, USA) according to manufacturer's recommendation. qPCR was performed on 1 ng of retro-transcribed product using TaqMan MicroRNA Assay primers for miR-210 and snoRNA-202 (Applied Biosystems), and the data were normalized to snoRNA-202.

### RNA extraction, reverse transcription and gene expression analysis

Total RNA was extracted from cell populations by the TRIzol method according to the manufacturer's instructions. cDNA was synthesized from 0.5-2 μg of total RNA using the reverse transcriptase (High Capacity cDNA Reverse Transcription Kit, Applied Biosystems). Each sample was run in triplicate on ABI PRISM^R^ 7900HT Sequence Detection System (PE Biosystems, Foster City, CA, USA). Results were analyzed with BNIP3 [22] and VEGF primer [53] using the comparative ΔΔCt method normalized to the housekeeping gene GAPDH. qPCR efficiency was in the range 95-105%.

### Western blot analysis

Cells were lysed in a buffer containing 140 mM NaCl, 20 mM Tris-HCl pH 7.4, 5 mM EDTA, 10% glycerol, 1% Triton X-100. Forty μg of cell lysate were loaded and subjected to SDS-PAGE and Western blotting (WB). The membranes were hybridized overnight with rabbit anti-mouse actin (1:5,000; Sigma-Aldrich) and rabbit anti-mouse HIF-1α (1:1,000; Novus Biologicals), followed by incubation with anti-rabbit HRP-conjugated antibody (1:5,000 dilution, Amersham-Pharmacia, Little Chalfont, U.K.). The signal was finally detected by chemiluminescence with SuperSignal kit (Pierce, Rockford, IL, USA) and lane densitometry analyzed by standard procedures [49].

### Statistical analysis

Data were analyzed using SigmaPlot software. Student's t-test, 2-way ANOVA statistical test were used as indicated. *P* values ≤ 0.05 were considered as significant.

## SUPPLEMENTARY FIGURES AND TABLES


