# Supplementary material for: A hypoxic signature marks tumors formed by disseminated tumor cells in the BALB-neuT mammary cancer model
Source: Oncotarget. 2016 Apr 20;7(22):33081–95. doi: 10.18632/oncotarget.8859 (PMC5078077; doi:10.18632/oncotarget.8859)
Supplement: Supplementary file 1 [file oncotarget-07-33081-s001.pdf]

A hypoxic signature marks tumors formed by disseminated tumor cells in the BALB-neuT mammary cancer model

SUPPLEMENTARY FIGURES AND TABLES

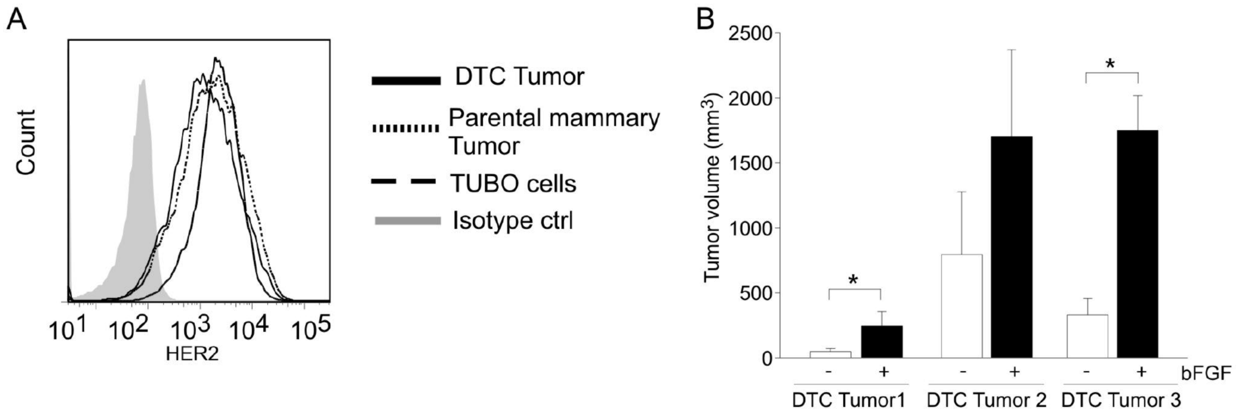

Supplementary Figure S1: Cells derived from DTC tumors express HER2 and generate secondary tumors whose growth is accelerated by the angiogenic factor bFGF. A. Flow cytometry analysis of HER2 expression in tumors generated by activation of late DTC (DTC tumor) compared to cells explanted from the parental BALB-neuT mammary tumor or TUBO cells. Grey shadow: non-specific negative control. **B.** Cell lines derived from three distinct DTC-tumors were s.c. inoculated into secondary recipient NSG mice with or without bFGF. Bars depict tumor volumes reached 28 days after cell injection. Data represent mean values (± SD). **P* < 0.05.

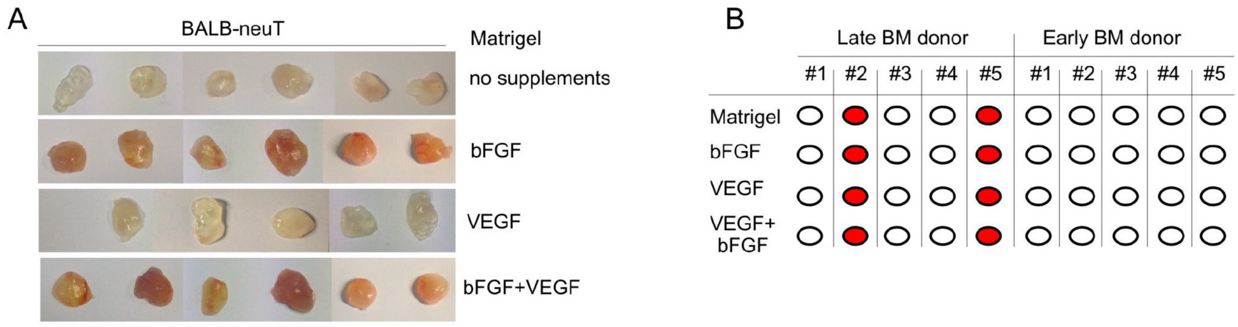

Supplementary Figure S2: Induction of angiogenesis by co-injection of BM cells with angiogenic factors and their effects on the outgrowth of DTC cells. A. Five million BM cells containing DTC were s.c. injected in Matrigel sponges with or without the angiogenic factors bFGF, VEGF or the combination of the two (500 ng of each factor per injection). One week later, Matrigel sponges were retrieved and photographed (n=5-6). **B.** BM cells were obtained from early and late donors (5 animals/group) and s.c. injected into NSG mice ($5x10^6$ cells/injection) in Matrigel alone or supplemented with bFGF, VEGF or the combination of the two (500 ng of each factor/injection). Red circles mark tumor formation; empty circles mark no tumor formation.

Supplementary Table S1: Outgrowth of late DTC recovered from individual BALB-neuT mice BM[a]

20-week-old late DTC donor mice		DTC-tumor formation in recipient NSG mice	
**Total tumor volume**	**Number of tumors**	**Number of DTC injected[b]**	**Tumor outgrowth**
775 mm^3	5	12	17 weeks
957 mm^3	4	21	26 weeks
239 mm^3	4	36	18 weeks
101 mm^3	2	90	11 weeks

a) Donors: 20-week-old BALB-neuT mice. Total tumor volume and numbers of tumors were evaluated before sacrifice of donors. The BM from these mice was harvested, and s.c. inoculated into NSG mice (5 x 10^6 BM-derived cells/site). Mice were inspected weekly and tumor scored when volume exceeded 10 mm^3.
b) DTC number was estimated by immunofluorescence analysis of CK8-18 and HER2 antigens.

Supplementary Table S2: DTC outgrowth is not affected by the angiogenic factor bFGF[a]

	Tumors/injected sites	Tumor take %
**No bFGF**	6/24	25 %
**With bFGF**	5/24	20 %

a) BM from old BALB-neuT mice were harvested and s.c. inoculated into NSG mice with or without bFGF (5×10^6 BM-derived cells/flank). Mice were inspected weekly for up to 30 weeks, and tumors scored when volume exceeded 10 mm^3.
